# Supplementary material for: Diverse Evolution in 111 Plant Genomes Reveals Purifying and Dosage Balancing Selection Models for F-Box Genes
Source: Int J Mol Sci. 2021 Jan 16;22(2):871. doi: 10.3390/ijms22020871 (PMC7829749; doi:10.3390/ijms22020871)

Supplemental Figure Legends

Figure S1. A bifurcated phylogenetic tree of 111 plant species. The order or plant group names are provided at each indicated node. The complete species names can be found in Table S1.

Figure S2. CTT annotation summary of *FBX* genes in 111 plant genomes. (A) A stepwise number comparison of sequences identified through the CTT annotation process. "Pseudo", "New" and "Prior" indicate *FBX* pseudogenes, newly annotated *FBX* loci and previously annotated *FBX* loci, respectively. (B) Correlation of the number of sequences identified in the different steps of the CTT annotation. (C) Correlation of genome size with the number of *FBX* loci in the three different groups listed in (A) in 111 plant species.

Figure S3. Statistical modelling of variation in the number of *FBX* genes in plant genomes. Black and cyan lines represent the empirical and expected data, respectively. The statistical model indicated in each panel was the best fitting model calculated using the "fitdistrplus" R package. The Kolmogorov–Smirnov test result is included in each panel to show the goodness-of-fit of the statistical model. Dashed red and green lines indicate the mean and mode number of *FBX* genes per genome, respectively. (A) Density curves of the number of *FBX* genes per plant genome in Clusters 1 and 2. (B) Density curves of the number of *FBX* genes per plant genome in Clusters 3 and 4.

Figure S4. Comparison of the lineage-specific expansion of *FBX* genes with total angiosperm gene families. (A) Number of *FBX* subfamilies in Clusters 1 and 4. (B) Number of lineage-specific and core angiosperm gene families.

Figure S5. Comparison of the number of different groups of *FBX* genes in 111 plant genomes. (A) Variation in the number of *FBX* gene superfamilies across 111 plant genomes. The solid blue line, gray shaded area, and bars with yellow, light green, orange, dark green, gray and cyan colors are as described in Figure 5. (B) Correlation of the number of orphan *FBX* genes with other groups of *FBX* genes in 111 plant genomes.

Figure S6. Statistical modeling of the number distribution of total, homologous and orphan *FBX* genes. Black and cyan lines represent the empirical and expected data, respectively. The statistical model indicated in each panel was the best fitting model calculated using the

“fitdistrplus” R package. The Kolmogorov–Smirnov test result is included in each panel to show the goodness-of-fit of the statistical model. Dashed red and green lines indicate the mean and mode number of *FBX* genes per genome, respectively. (A) Total number of *FBX* genes per genome. (B) Number of homologous *FBX* genes per genome. (C) Number of orphan *FBX* genes per genome.

Figure S7. Analysis of the role of three WGD events in contributing to the complete set of *FBX* duplications in 27 flowering plants. The species belonging to five groups of flowering plants are labeled with one character as follows, B: Brassicales; G: *Gossypium raimondii* (*Grai*); M: *Musa acuminata* (*Macu*); P: Poaceae; R: Rosids. The result of χ^2 goodness-of-fit data is provided to demonstrate the fitness of the observed data to the indicated power-law curve.

Figure S8. Analysis of the role of three WGD events in contributing to *FBX* duplications in four different clusters in 27 flowering plants. The letter code for the five groups of flowering plants is as indicated in Figure S7. The result of χ^2 goodness-of-fit data is provided in each panel to demonstrate the fitness of the observed data to the indicated power-law curve. The four clusters of FBX genes are described in Figure 3A. (A) Cluster 1. (B) Cluster 2. (C) Cluster 3. (D) Cluster 4.

Figure S9. Differential contribution of homologous and orphan loci in expanding the size of the *FBX* gene superfamily in 111 plant genomes. The solid blue line, gray shaded area, and bars with yellow, light green, orange, dark green, gray and cyan colors are as described in Figure 5. (A) Proportion of the total number of homologous *FBX* genes per genome. (B) Proportion of the total number of orphan *FBX* genes per genome. (C) Proportion of Cluster 1 homologous *FBX* genes per genome. (D) Proportion of Cluster 2 homologous *FBX* genes per genome. (E) Proportion of Cluster 3 homologous *FBX* genes per genome. (F) Proportion of Cluster 4 homologous *FBX* genes per genome.

Figure S10. Correlation of the species tree matrix with *FBX* subfamily sizes for the four clusters of *FBX* genes in each plant genome. The species matrix was converted from the species tree using the “phytools” R package. The dendrogram of each data matrix was created using the “dist” (method = "manhattan") and “hclust” (method = "ward.D2") functions in the “gplots” R package. The spearman’s dendrogram correlation was calculated using the “dendextend” R package.

Figure S11. Statistical modeling test of SCPs in Cluster 4 subfamilies. Three different statistical models constructed using the "fitdistrplus" R package are shown to model the distribution of SCP values. (A) The histogram of observed data is plotted against three fitted density functions. (B) The empirical cumulative distribution (black dots) is plotted against three fitted distribution functions. (C) The empirical quantiles are plotted as functions of theoretical quantiles of three fitted models. (D) The empirical probabilities are plotted as functions of theoretical probabilities of three fitted models. The Kolmogorov–Smirnov test results are included to show the goodness-of-fit of three statistical models in (B).

Figure S12. A normal distribution of SCPs in Cluster 3 subfamilies. The model was constructed using the "fitdistrplus" R package. Black and red lines indicate the empirical and expected distribution of SCP values, respectively. The Kolmogorov–Smirnov test result is shown to demonstrate the goodness-of-fit of the model.

Figure S13. An enlarged view showing the *hc* clustering of Cluster 4 *FBX* subfamilies and the interaction of CAFs with ASK1. The CAF proteins demonstrated to interact with ASK1 in previous literature or in this work are shown blue and red, respectively. The untested subfamilies are highlighted in black. The asterisks indicate CAF proteins that have been confirmed to interact with ASK1 in both previous literature and this work. The two arrowheads indicate subfamilies that are absent in Arabidopsis. The color code of the side bar is as described in Figure 8A. The accession number of each CAF protein, and the corresponding references describing their interactions with ASK1, can be found in Table S2.

Figure S14. Enrichment assay of known Arabidopsis *FBX* genes in four different clusters. The *p*-values were calculated based on Fisher's exact test for the indicated pairs of datasets.

Hua, 2020; Figure S1

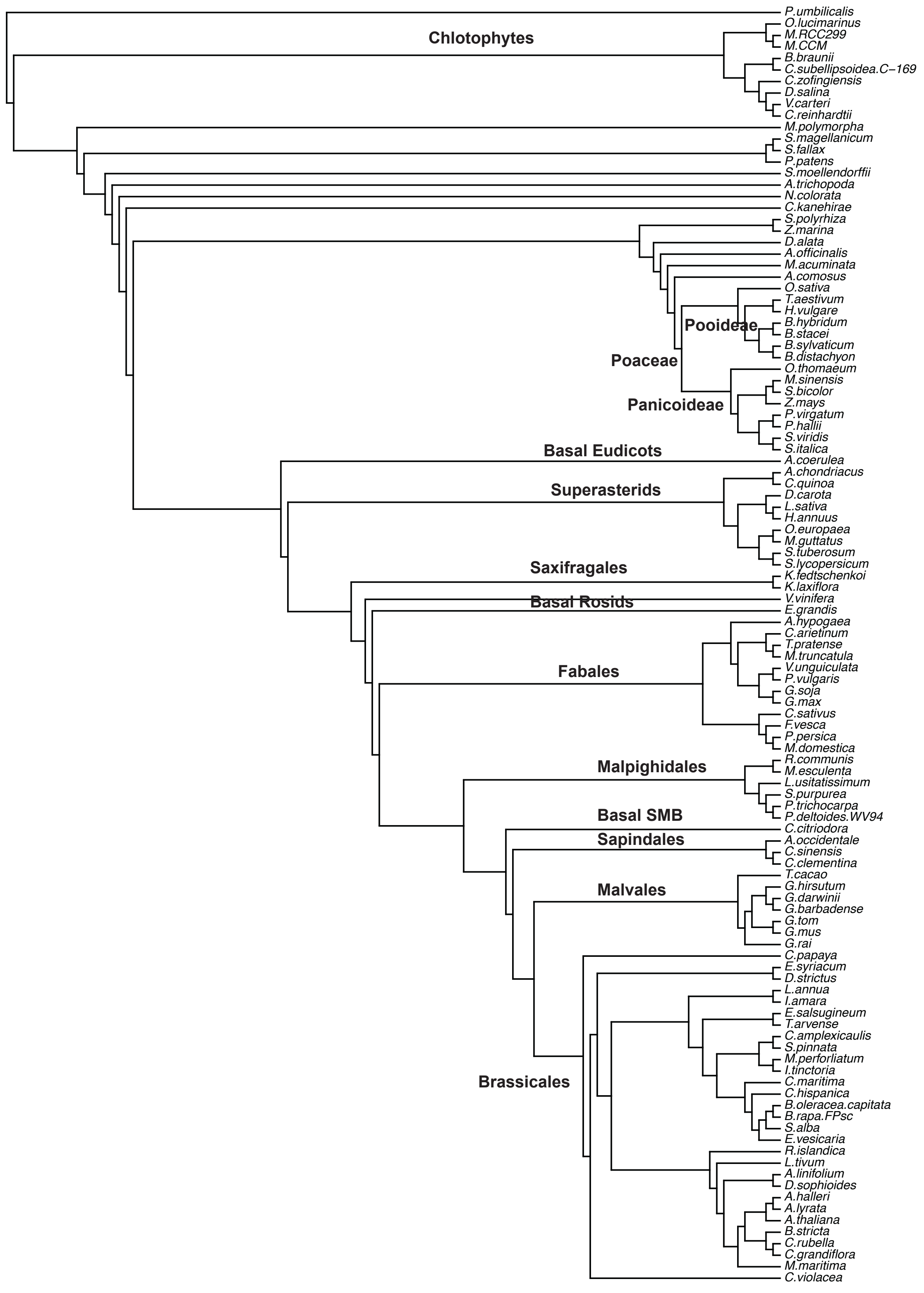

Hua, 2020; Figure S2

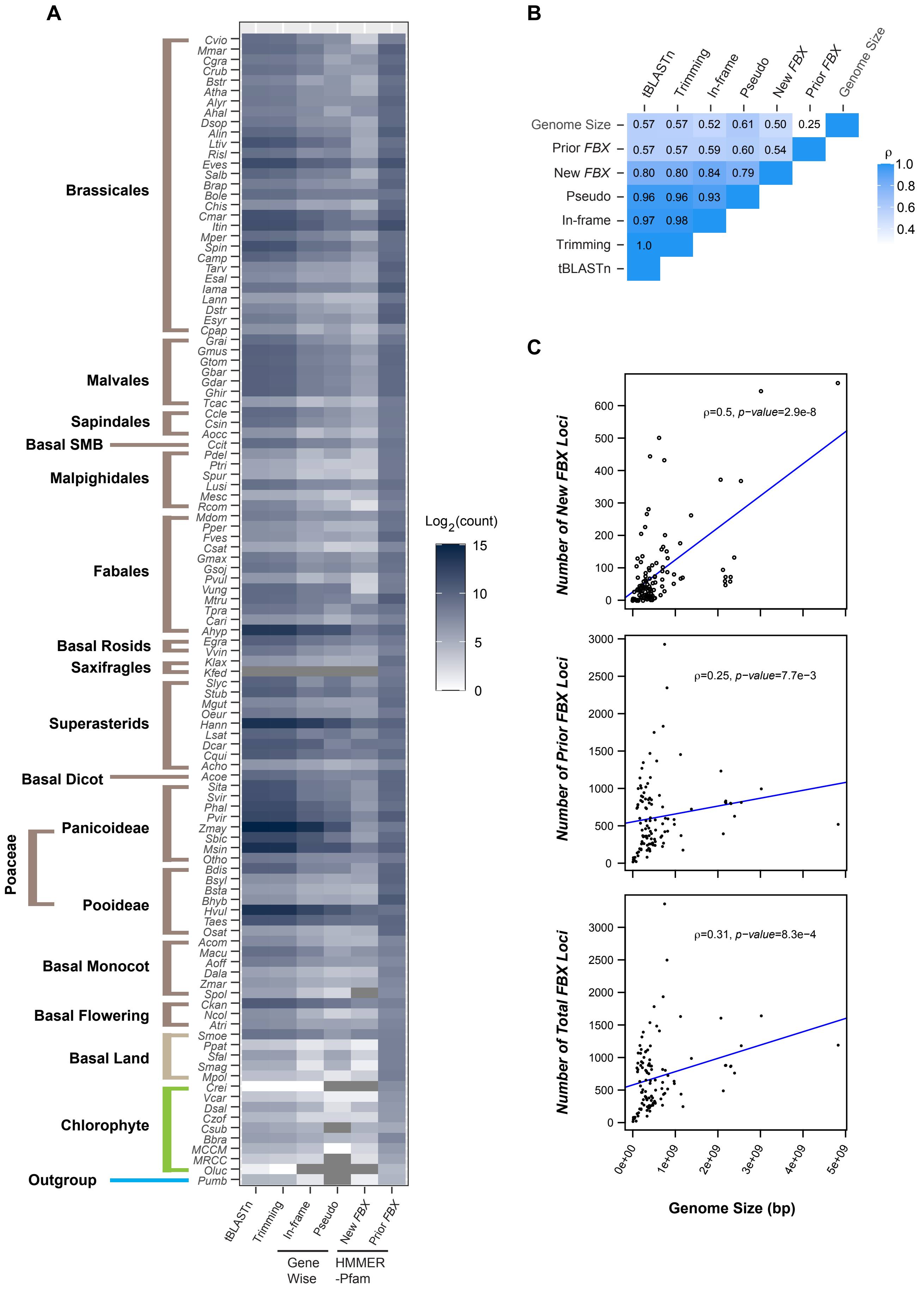

Hua, 2020; Figure S3

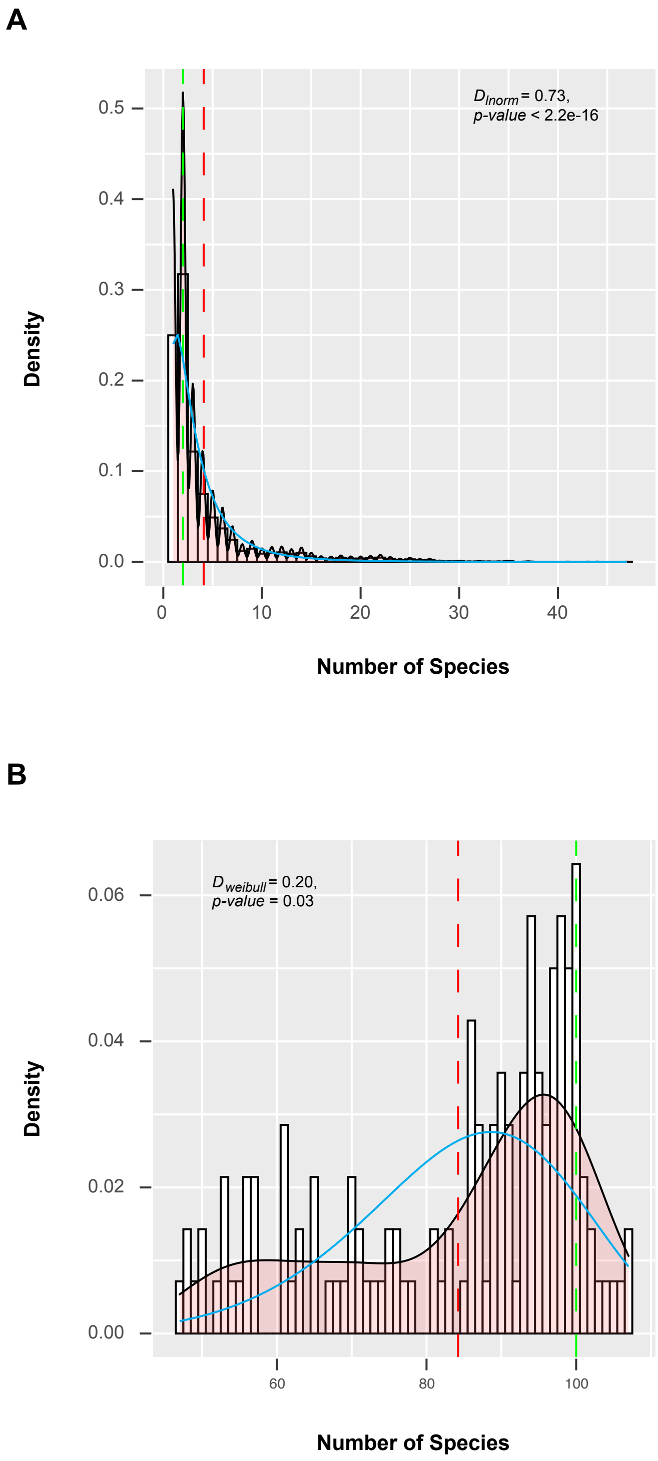

Hua, 2020; Figure S4

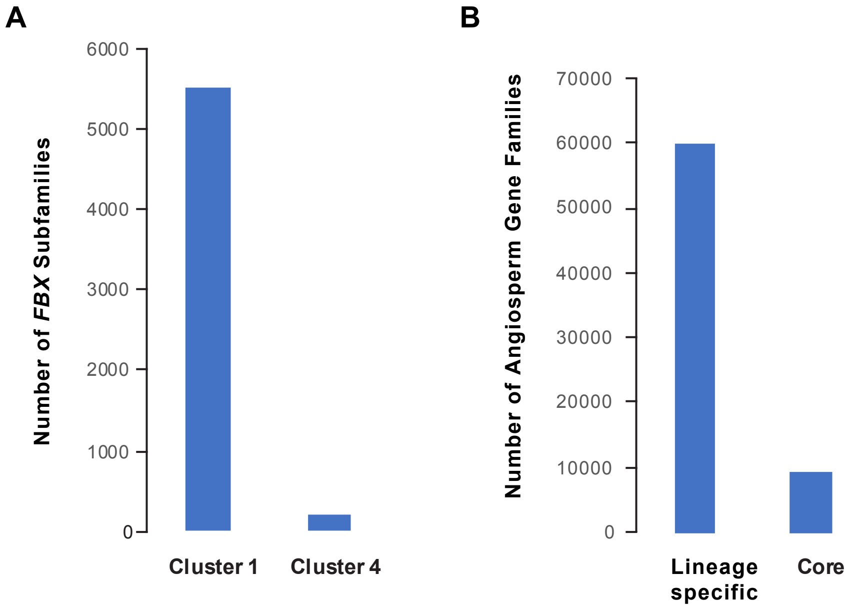

Hua, 2020; Figure S5

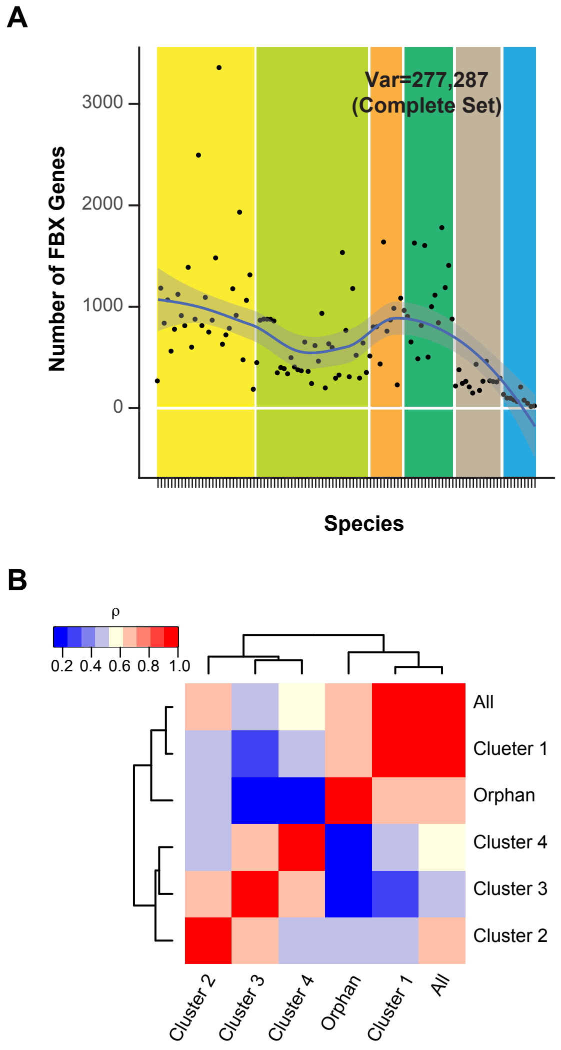

Hua, 2020; Figure S6

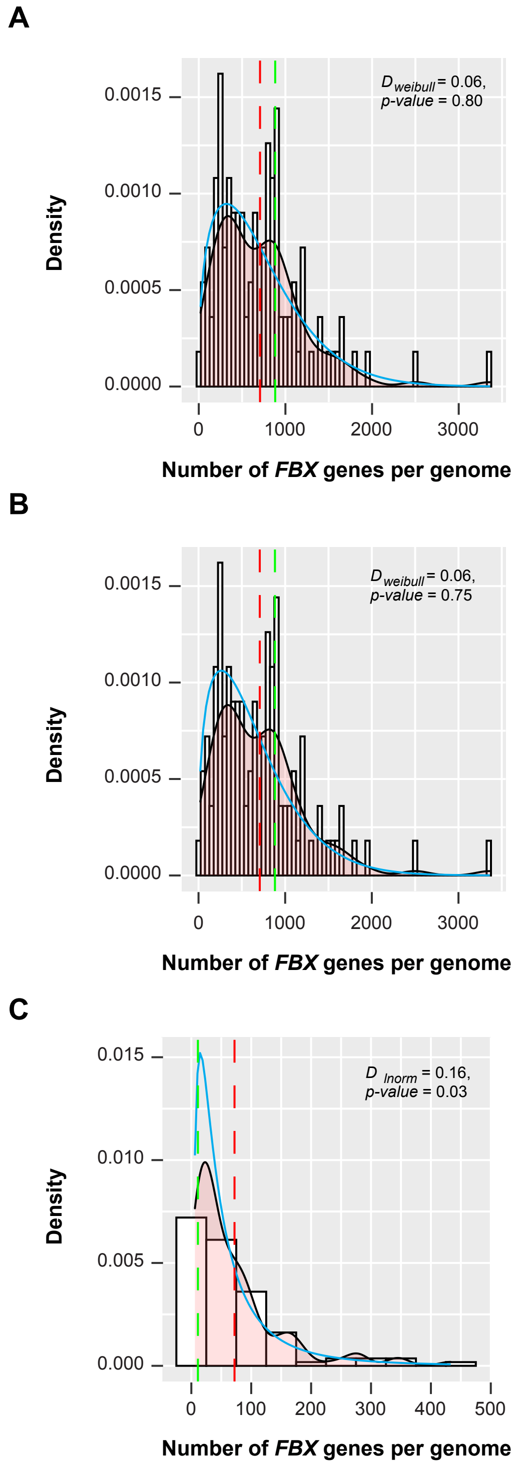

Hua, 2020; Figure S7

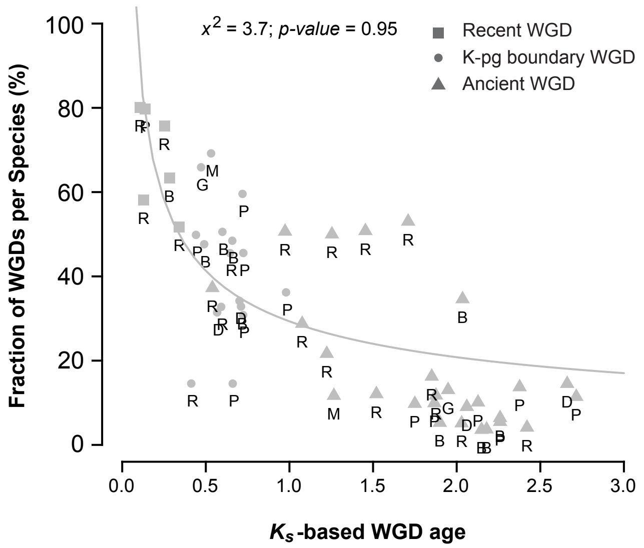

Hua, 2020; Figure S8

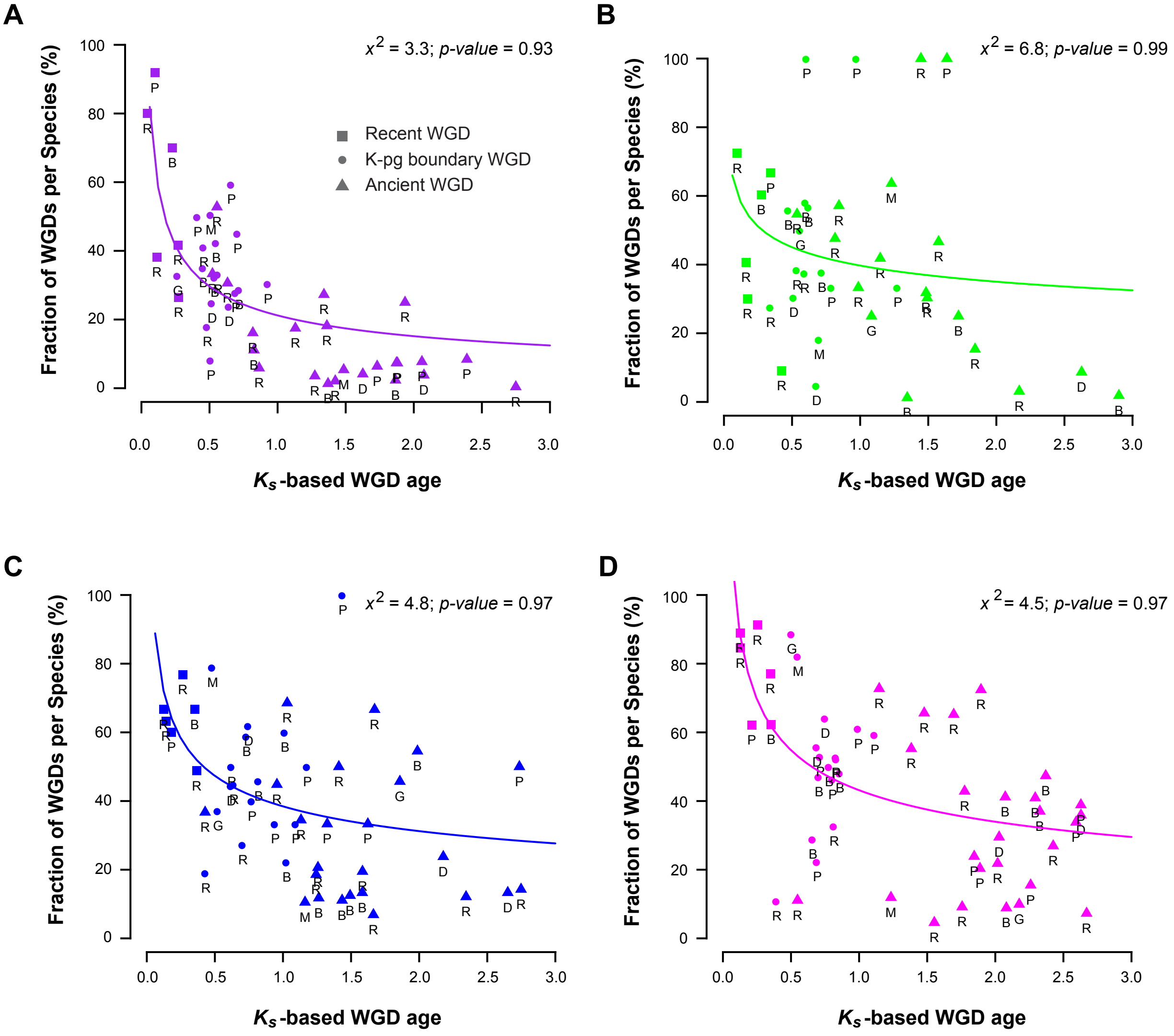

Hua, 2020; Figure S9

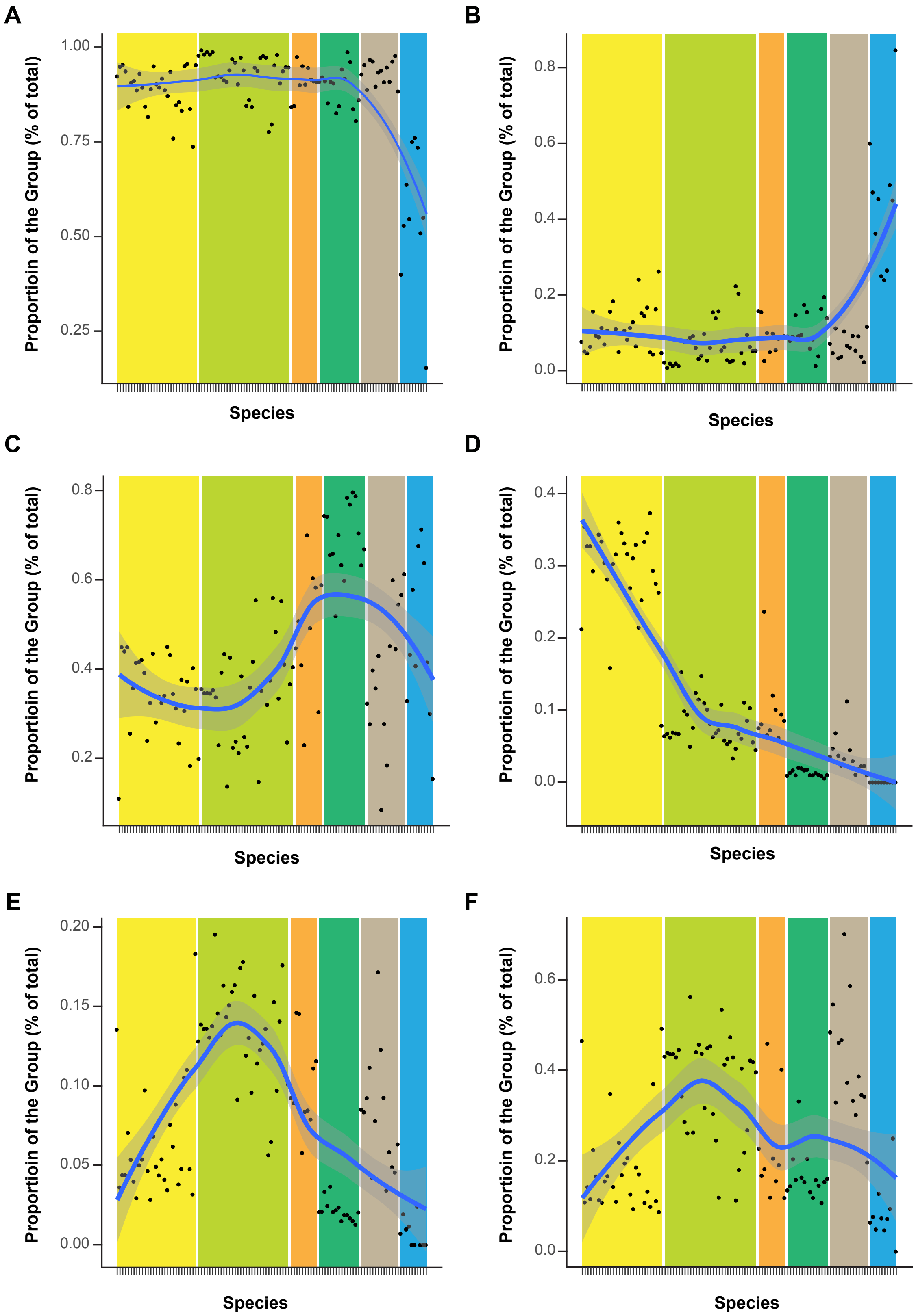

Hua, 2020; Figure S10

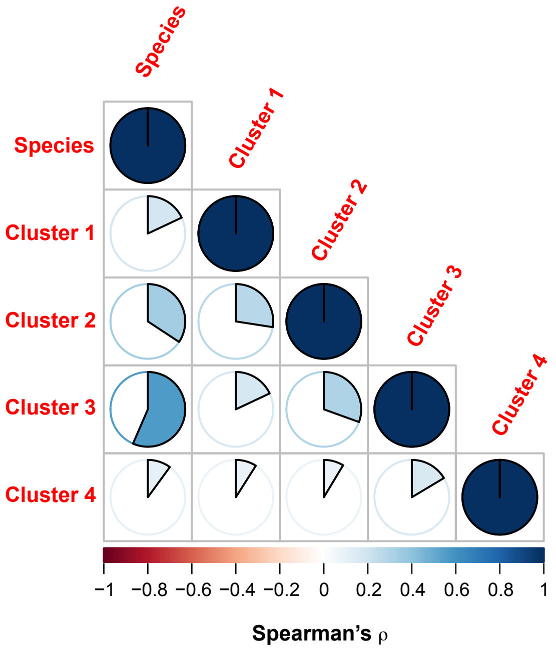

Hua, 2020; Figure S11

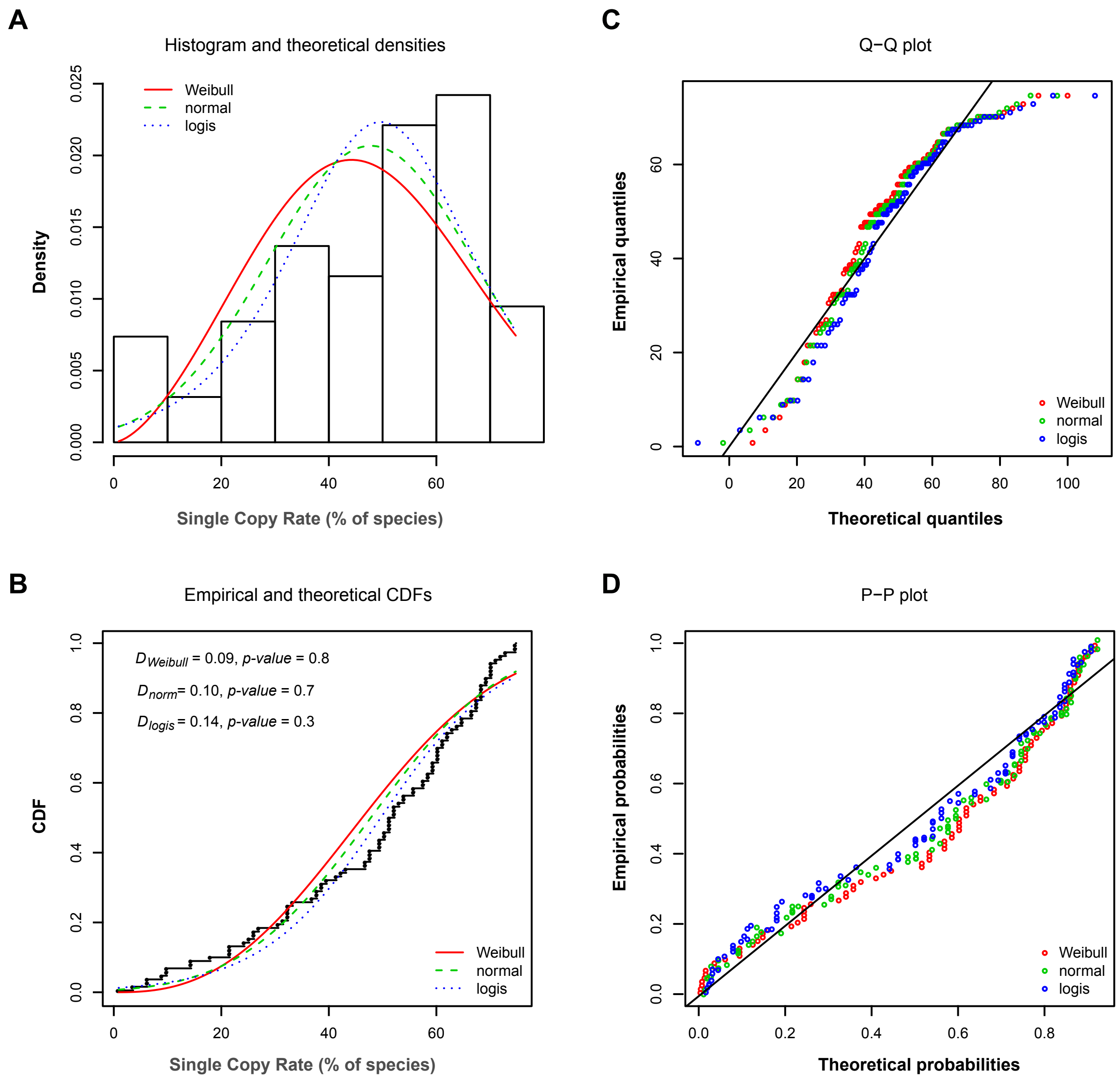

Hua, 2020; Figure S12

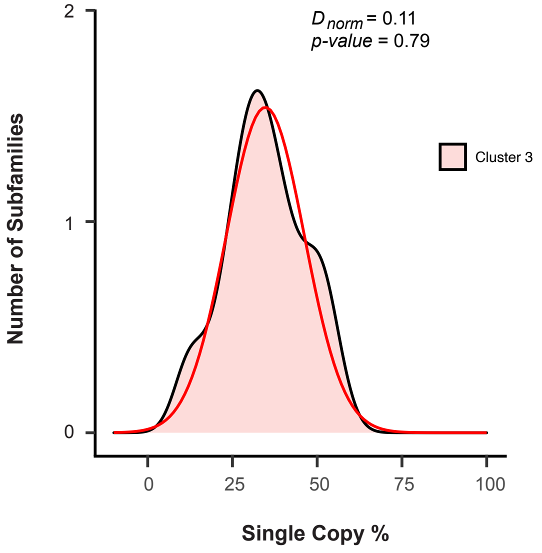

Hua, 2020; Figure S13

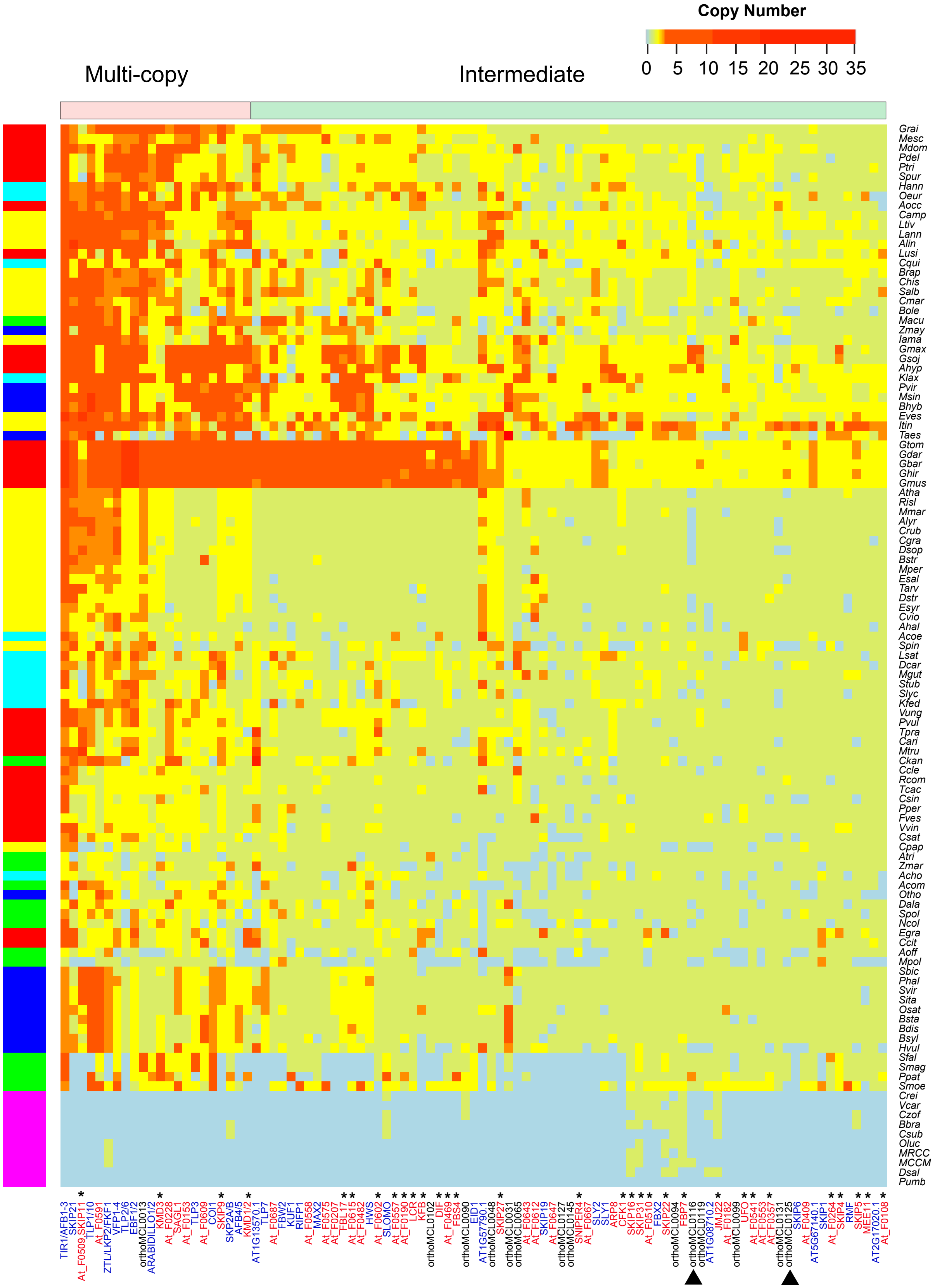

Hua, 2020; Figure S14

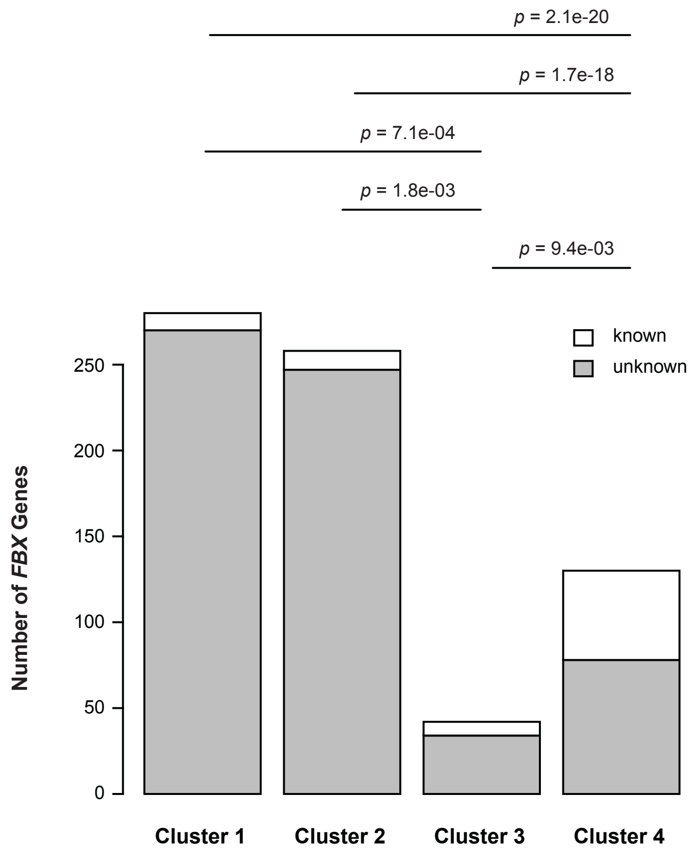

Supplement: Supplementary file 1 [file ijms-22-00871-s001.zip › Supplementary Materials/supplementary Figures.pdf]
