# Supplementary material for: Diverse Evolution in 111 Plant Genomes Reveals Purifying and Dosage Balancing Selection Models for F-Box Genes
Source: Int J Mol Sci. 2021 Jan 16;22(2):871. doi: 10.3390/ijms22020871 (PMC7829749; doi:10.3390/ijms22020871)
Supplement: Supplementary file 1 [file ijms-22-00871-s001.zip › Supplementary Materials/Table S2. Biochemical interaction analysis of CAFs with ASK1.pdf]

Table S2. Biochemical interaction analysis of CAFs with ASK1

Subfamily	ID	AGI	Known Name	Y2H_Clone (ID in (Hua, et al. 2011))	ASK1 Interaction	Reference
orthoMCL0000	AthaFBX_00557	AT3G26810.1	AFB2		Y	(Dharmasiri, et al. 2005)
orthoMCL0000	AthaFBX_00576	AT3G62980.1	TIR1		Y	(Gray, et al. 1999; Dharmasiri, et al. 2005)
orthoMCL0000	AthaFBX_00617	AT1G12820.1	AFB3		Y	(Dharmasiri, et al. 2005)
orthoMCL0000	AthaFBX_00626	AT4G03190.1	GRH1		Y	(Dharmasiri, et al. 2005)
orthoMCL0003	AthaFBX_00002	AT1G76900.1	TLP1		Y	(Kuroda, et al. 2012; Bao, et al. 2014)
orthoMCL0003	AthaFBX_00012	AT1G25280.1	TLP10		Y	(Risseeuw, et al. 2003; Kuroda, et al. 2012; Bao, et al. 2014)
orthoMCL0003	AthaFBX_00071	AT1G43640.1	TLP5			
orthoMCL0005	AthaFBX_00231	AT5G25350.1	EBF2		Y	(Guo and Ecker 2003; Potuschak, et al. 2003; Gagne, et al. 2004; Kuroda, et al. 2012)
orthoMCL0005	AthaFBX_00332	AT2G25490.1	EBF1		Y	(Guo and Ecker 2003; Potuschak, et al. 2003; Gagne, et al. 2004; Kuroda, et al. 2012)
orthoMCL0006	AthaFBX_00134	AT5G67250.1	VFB4		Y	(van den Burg, et al. 2008; Arabidopsis Interactome Mapping 2011)
orthoMCL0006	AthaFBX_00178	AT3G50080.1	VFB2		Y	(van den Burg, et al. 2008)
orthoMCL0006	AthaFBX_00199	AT4G07400.1	VFB3		Y	(van den Burg, et al. 2008)
orthoMCL0006	AthaFBX_00409	AT1G47056.1	VFB1		Y	(van den Burg, et al. 2008)
orthoMCL0007	AthaFBX_00266	AT5G57360.2	ZTL		Y	(Han, et al. 2004)
orthoMCL0007	AthaFBX_00448	AT2G18915.2	LKP2		Y	(Yasuhara, et al. 2004)
orthoMCL0007	AthaFBX_00578	AT1G68050.1	FKF1		Y	(Yasuhara, et al. 2004; Arabidopsis Interactome Mapping 2011)
orthoMCL0008	AthaFBX_00746	AT2G02870.1	SKIP11	At_F0678	Y	(Risseeuw, et al. 2003; Arabidopsis Interactome Mapping 2011)
orthoMCL0008	AthaFBX_00533	AT1G74510.2		At_F0509	Y	
orthoMCL0008	AthaFBX_00687	AT1G26930.1			Y	(Arabidopsis Interactome Mapping 2011)
orthoMCL0009	AthaFBX_00587	AT4G18380.1				
orthoMCL0009	AthaFBX_00666	AT5G46170.1		At_F0591	Y	
orthoMCL0009	AthaFBX_00655	AT1G30200.2				
orthoMCL0010	AthaFBX_00219	AT2G02240.1	MEE66			
orthoMCL0010	AthaFBX_00321	AT2G02360.1	PP2-B10		Y	(Arabidopsis Interactome Mapping 2011)
orthoMCL0010	AthaFBX_00382	AT2G02230.1	SKIP21 / PP2-B1		Y	(Risseeuw, et al. 2003; Kuroda, et al. 2012)

orthoMCL0010	AthaFBX_00118	AT2G02250.1	PP2-B2			
orthoMCL0013	AthaFBX_00620	AT5G07670.1				
orthoMCL0013	AthaFBX_00648	AT5G51380.1				
orthoMCL0013	AthaFBX_00629	AT5G51370.2				
orthoMCL0014	AthaFBX_00434	AT1G55270.1	SAGL1	At_F0445	Y	
orthoMCL0015	AthaFBX_00202	AT1G47270.1	TLP6		Y	(Kuroda, et al. 2012)
orthoMCL0015	AthaFBX_00399	AT2G18280.2	TLP2		Y	(Kuroda, et al. 2012)
orthoMCL0017	AthaFBX_00615	AT3G61060.2	SKIP9 / PP2-A13	At_F0468	Y	(Risseeuw, et al. 2003)
orthoMCL0017	AthaFBX_00599	AT1G12710.2	PP2-A12	At_F0514	Y	(Hua and Gao 2019)
orthoMCL0018	AthaFBX_00694	AT2G39940.1	COI1		Y	(Xu, et al. 2002; Takahashi, et al. 2004)
orthoMCL0019	AthaFBX_00189	AT2G47900.3	TLP3		Y	(Kuroda, et al. 2012; Bao, et al. 2014)
orthoMCL0020	AthaFBX_00350	AT2G44130.1	KMD3	At_F0354	Y	(Kim, et al. 2013; Hua and Gao 2019)
orthoMCL0020	AthaFBX_00613	AT3G59940.1	KMD4		Y	(Kim, et al. 2013) (Risseeuw, et al. 2003; Arabidopsis Interactome Mapping 2011)
orthoMCL0021	AthaFBX_00400	AT1G13570.1			Y	(Kuroda, et al. 2012)
orthoMCL0023	AthaFBX_00194	AT2G26850.1		At_F0228	Y	
orthoMCL0023	AthaFBX_00412	AT2G32560.1		At_F0365	?	
orthoMCL0024	AthaFBX_00250	AT1G21410.1	SKP2A		Y	(Jurado, et al. 2008; Kuroda, et al. 2012)
orthoMCL0024	AthaFBX_00480	AT1G77000.1	SKP2B		Y	(Kuroda, et al. 2012)
orthoMCL0025	AthaFBX_00481	AT3G60350.1	ARABIDILLO-2		Y	(Nibau, et al. 2011)
orthoMCL0025	AthaFBX_00601	AT2G44900.2	ARABIDILLO-1		Y	(Nibau, et al. 2011)
orthoMCL0027	AthaFBX_00635	AT4G00755.1		At_F0609	Y	
orthoMCL0028	AthaFBX_00511	AT1G57790.1			Y	(Kuroda, et al. 2012)
orthoMCL0028	AthaFBX_00278	AT3G56470.1				
orthoMCL0028	AthaFBX_00523	AT4G12382.1				
orthoMCL0030	AthaFBX_00394	AT1G22040.1		At_F0153	Y	
orthoMCL0031	AthaFBX_00659	AT1G80570.2				
orthoMCL0034	AthaFBX_00642	AT1G80440.1	KMD1	At_F0564	Y	(Kim, et al. 2013)
orthoMCL0034	AthaFBX_00730	AT1G15670.1	KMD2	At_F0656	Y	(Kim, et al. 2013)
orthoMCL0035	AthaFBX_00696	AT1G67340.1		At_F0687	Y	
orthoMCL0036	AthaFBX_00038	AT3G61590.4	HWS		Y	(Takahashi, et al. 2004; Gonzalez-Carranza, et al. 2007)
orthoMCL0037	AthaFBX_00639	AT5G49980.1	AFB5		Y	(Prigge, et al. 2016)
orthoMCL0037	AthaFBX_00649	AT4G24390.1	AFB4		Y	(Prigge, et al. 2016)
orthoMCL0039	AthaFBX_00704	AT1G61340.1	FBS1		Y	(Arabidopsis Interactome Mapping 2011; Sepulveda-Garcia and Rocha-Sosa 2012)
orthoMCL0039	AthaFBX_00668	AT4G21510.1	SKIP27 / FBS2	At_F0559	Y	(Risseeuw, et al. 2003)
orthoMCL0042	AthaFBX_00424	AT4G33210.1	SLOMO		Y	(Lohmann, et al. 2010)
orthoMCL0043	AthaFBX_00604	AT1G67480.1		At_F0482	?	
orthoMCL0044	AthaFBX_00637	AT3G26000.1	RIFP1		Y	(Risseeuw, et al. 2003; Li, et al. 2016)

orthoMCL0045	AthaFBX_00509	AT1G53320.1	TLP7		Y	(Bao, et al. 2014)
orthoMCL0047	AthaFBX_00471	AT1G10780.2		At_F0615	Y	(Kuroda, et al. 2012)
orthoMCL0048	AthaFBX_00701	AT5G39360.1	EDL2			
orthoMCL0048	AthaFBX_00722	AT5G15440.1	EDL1			
orthoMCL0049	AthaFBX_00376	AT3G54650.1	FBL17	At_F0563	Y	(Kim, et al. 2008; Hua and Gao 2019)
orthoMCL0050	AthaFBX_00505	AT4G08980.2	FBW2	At_F0505	Y	(Kuroda, et al. 2012)
orthoMCL0051	AthaFBX_00544	AT1G67190.2		At_F0602	Y	(Kuroda, et al. 2012)
orthoMCL0052	AthaFBX_00714	AT2G42620.1	MAX2		Y	(Woo, et al. 2001; Stirnberg, et al. 2007)
orthoMCL0054	AthaFBX_00606	AT3G63220.2		At_F0557	Y	(Risseeuw, et al. 2003)
orthoMCL0055	AthaFBX_00203	AT1G27340.1	LCR	At_F0313	Y	(Hua and Gao 2019)
orthoMCL0056	AthaFBX_00612	AT4G10925.4		At_F0558	?	
orthoMCL0057	AthaFBX_00237	AT5G39450.1		At_F0202	Y	
orthoMCL0057	AthaFBX_00644	AT5G39490.1		At_F0607	Y	
orthoMCL0057	AthaFBX_00677	AT5G39460.1		At_F0643	Y	
orthoMCL0059	AthaFBX_00679	AT1G31350.2	KUF1		Y	(Risseeuw, et al. 2003)
orthoMCL0061	AthaFBX_00174	AT4G33160.2		At_F0207	Y	
orthoMCL0063	AthaFBX_00512	AT1G23390.1	KFB	At_F0500	Y	(Kuroda, et al. 2012; Zhang, et al. 2013; Hua and Gao 2019)
orthoMCL0065	AthaFBX_00699	AT2G27310.1				
orthoMCL0066	AthaFBX_00293	AT4G24210.1	SLY1		Y	(Fu, et al. 2004)
orthoMCL0067	AthaFBX_00619	AT5G04010.1		At_F0612	Y	
orthoMCL0068	AthaFBX_00614	AT5G60570.2		At_F0575	Y	
orthoMCL0069	AthaFBX_00137	AT5G45360.1	SKIP31	At_F0211	Y	(Risseeuw, et al. 2003; Kuroda, et al. 2012)
orthoMCL0070	AthaFBX_00285	AT5G48170.1	SLY2		Y	(Fu, et al. 2004)
orthoMCL0071	AthaFBX_00213	AT1G23780.1	SKIP22	At_F0283	Y	(Risseeuw, et al. 2003; Kuroda, et al. 2012)
orthoMCL0074	AthaFBX_00586	AT4G02440.1	EID1		Y	(Dieterle, et al. 2001; Risseeuw, et al. 2003; Arabidopsis Interactome Mapping 2011)
orthoMCL0075	AthaFBX_00031	AT1G21760.1	FBP7	At_F0001	Y	(Risseeuw, et al. 2003; Calderon-Villalobos, et al. 2007; Kuroda, et al. 2012; Hua and Gao 2019)
orthoMCL0077	AthaFBX_00377	AT1G70590.1		At_F0469	Y	(Kuroda, et al. 2012)
orthoMCL0078	AthaFBX_00553	AT1G30090.1	DIF	At_F0466	Y	(Hua and Gao 2019)
orthoMCL0080	AthaFBX_00245	AT5G15710.2		At_F0190	Y	(Kuroda, et al. 2012)
orthoMCL0081	AthaFBX_00403	AT5G21040.2	FBX2		Y	(Kuroda, et al. 2012)
orthoMCL0083	AthaFBX_00744	AT4G35930.3	FBS4	At_F0709	Y	(Hua and Gao 2019)
orthoMCL0085	AthaFBX_00729	AT1G06110.1	SKIP16	At_F0664	Y	(Hua and Gao 2019)
orthoMCL0086	AthaFBX_00146	AT5G42350.1	CFK1	At_F0220	Y	(Hua and Gao 2019)
orthoMCL0086	AthaFBX_00149	AT5G42360.1	CFK2			
orthoMCL0088	AthaFBX_00393	AT1G51550.1		At_F0357	Y	(Kuroda, et al. 2012)

orthoMCL0090	AthaFBX_00521	AT1G78280.1				
orthoMCL0091	AthaFBX_00380	AT5G56180.1	ARP8	At_F0323	Y	
orthoMCL0092	AthaFBX_00345	AT3G61350.2	SKIP4	At_F0437	Y	(Hua and Gao 2019)
orthoMCL0093	AthaFBX_00570	AT1G55000.1		At_F0510	Y	(Arabidopsis Interactome Mapping 2011)
orthoMCL0094	AthaFBX_00478	AT3G54460.1				
orthoMCL0095	AthaFBX_00561	AT5G67140.1			Y	(Kuroda, et al. 2012)
orthoMCL0096	AthaFBX_00191	AT3G54480.1	SKIP5	At_F0362	Y	(Kuroda, et al. 2012)
orthoMCL0097	AthaFBX_00698	AT5G36000.1				
orthoMCL0097	AthaFBX_00705	AT3G61730.1	RMF		Y	(Kim, et al. 2010)
orthoMCL0098	AthaFBX_00379	AT1G30950.1	UFO	At_F0267	Y	(Durfee, et al. 2003; Kuroda, et al. 2012; Hua and Gao 2019)
orthoMCL0099	AthaFBX_00715	AT5G23340.1				
orthoMCL0100	AthaFBX_00132	AT4G05460.1	SKIP19		Y	(Kuroda, et al. 2012)
orthoMCL0102	AthaFBX_00695	AT5G40470.1				
orthoMCL0103	AthaFBX_00342	AT3G52030.1		At_F0264	Y	(Kuroda, et al. 2012)
orthoMCL0104	AthaFBX_00690	AT5G57900.1	SKIP1		Y	(Risseeuw, et al. 2003)
orthoMCL0105	AthaFBX_00624	AT5G06550.1	JMJ22	At_F0649	Y	(Hua and Gao 2019)
orthoMCL0106	AthaFBX_00580	AT4G03030.1		At_F0553	Y	
orthoMCL0110	AthaFBX_00467	AT3G48880.3	SNIPER4	At_F0538	Y	(Arabidopsis Interactome Mapping 2011; Kuroda, et al. 2012; Huang, et al. 2018)
orthoMCL0114	AthaFBX_00406	AT5G39250.1		At_F0541	Y	(Kuroda, et al. 2012)
orthoMCL0117	AthaFBX_00674	AT2G36090.1		At_F0667	Y	
orthoMCL0118	AthaFBX_00057	AT5G52880.1		At_F0108	Y	(Kuroda, et al. 2012)
orthoMCL0119	AthaFBX_00459	AT4G15475.1	RAD7B			
orthoMCL0120	AthaFBX_00466	AT3G12350.1		At_F0409	Y	
orthoMCL0121	AthaFBX_00652	AT2G01620.1	MEE11	At_F0710	Y	(Risseeuw, et al. 2003; Arabidopsis Interactome Mapping 2011; Hua and Gao 2019)
orthoMCL0123	AthaFBX_00284	AT2G17020.1			Y	(Kuroda, et al. 2012)
orthoMCL0126	AthaFBX_00532	AT2G21950.1	SKIP6		Y	(Risseeuw, et al. 2003)
orthoMCL0127	AthaFBX_00158	AT1G76920.1				
orthoMCL0131	AthaFBX_00708	AT3G20270.3				
orthoMCL0136	AthaFBX_00750	AT1G08710.2	SKIP24		Y	(Risseeuw, et al. 2003)
orthoMCL0138	AthaFBX_00159	AT5G26960.1		At_F0182	Y	
orthoMCL0144	AthaFBX_00691	AT3G07550.2		At_F0647	Y	
orthoMCL0145	AthaFBX_00638	AT5G19970.1				

References

Arabidopsis Interactome Mapping Consortium. 2011. Evidence for network evolution in an Arabidopsis interactome map. Science 333:601-607.

Bao Y, Song WM, Jin YL, Jiang CM, Yang Y, Li B, Huang WJ, Liu H, Zhang HX. 2014. Characterization of Arabidopsis Tubby-like proteins and redundant function of AtTLP3 and AtTLP9 in plant response to ABA and osmotic stress. Plant Mol Biol 86:471-483.

Calderon-Villalobos LI, Nill C, Marrocco K, Kretsch T, Schwechheimer C. 2007. The evolutionarily conserved Arabidopsis thaliana F-box protein AtFBP7 is required for efficient translation during temperature stress. Gene 392:106-116.
Dharmasiri N, Dharmasiri S, Weijers D, Lechner E, Yamada M, Hobbie L, Ehrismann JS, Jurgens G, Estelle M. 2005. Plant development is regulated by a family of auxin receptor F box proteins. Dev Cell 9:109-119.
Dieterle M, Zhou YC, Schafer E, Funk M, Kretsch T. 2001. EID1, an F-box protein involved in phytochrome A-specific light signaling. Genes Dev 15:939-944.
Durfee T, Roe JL, Sessions RA, Inouye C, Serikawa K, Feldmann KA, Weigel D, Zambryski PC. 2003. The F-box-containing protein UFO and AGAMOUS participate in antagonistic pathways governing early petal development in Arabidopsis. Proc Natl Acad Sci U S A 100:8571-8576.
Fu X, Richards DE, Fleck B, Xie D, Burton N, Harberd NP. 2004. The Arabidopsis mutant sleepy1gar2-1 protein promotes plant growth by increasing the affinity of the SCFSLY1 E3 ubiquitin ligase for DELLA protein substrates. Plant Cell 16:1406-1418.
Gagne JM, Smalle J, Gingerich DJ, Walker JM, Yoo SD, Yanagisawa S, Vierstra RD. 2004. Arabidopsis EIN3-binding F-box 1 and 2 form ubiquitin-protein ligases that repress ethylene action and promote growth by directing EIN3 degradation. Proc Natl Acad Sci U S A 101:6803-6808.
Gonzalez-Carranza ZH, Rompa U, Peters JL, Bhatt AM, Wagstaff C, Stead AD, Roberts JA. 2007. Hawaiian skirt: an F-box gene that regulates organ fusion and growth in Arabidopsis. Plant Physiol 144:1370-1382.
Gray WM, del Pozo JC, Walker L, Hobbie L, Risseeuw E, Banks T, Crosby WL, Yang M, Ma H, Estelle M. 1999. Identification of an SCF ubiquitin-ligase complex required for auxin response in Arabidopsis thaliana. Genes Dev 13:1678-1691.
Guo H, Ecker JR. 2003. Plant responses to ethylene gas are mediated by $SCF^{EBF1/EBF2}$-dependent proteolysis of EIN3 transcription factor. Cell 115:667-677.
Han L, Mason M, Risseeuw EP, Crosby WL, Somers DE. 2004. Formation of an SCF(ZTL) complex is required for proper regulation of circadian timing. Plant J 40:291-301.
Hua Z, Gao Z. 2019. Adaptive and degenerative evolution of the S-Phase Kinase-Associated Protein 1-Like family in Arabidopsis thaliana. PeerJ 7:e6740.
Hua Z, Zou C, Shiu SH, Vierstra RD. 2011. Phylogenetic comparison of *F-Box* (*FBX*) gene superfamily within the plant kingdom reveals divergent evolutionary histories indicative of genomic drift. PLoS One 6:e16219.
Huang J, Zhu C, Li X. 2018. SCF(SNIPER4) controls the turnover of two redundant TRAF proteins in plant immunity. Plant J 95:504-515.
Jurado S, Diaz-Trivino S, Abraham Z, Manzano C, Gutierrez C, del Pozo C. 2008. SKP2A, an F-box protein that regulates cell division, is degraded via the ubiquitin pathway. Plant J 53:828-841.
Kim HJ, Chiang YH, Kieber JJ, Schaller GE. 2013. SCF(KMD) controls cytokinin signaling by regulating the degradation of type-B response regulators. Proc Natl Acad Sci U S A 110:10028-10033.
Kim HJ, Oh SA, Brownfield L, Hong SH, Ryu H, Hwang I, Twell D, Nam HG. 2008. Control of plant germline proliferation by SCF^{FBL17} degradation of cell cycle inhibitors. Nature 455:1134-1137.
Kim OK, Jung JH, Park CM. 2010. An Arabidopsis F-box protein regulates tapetum degeneration and pollen maturation during anther development. Planta 232:353-366.
Kuroda H, Yanagawa Y, Takahashi N, Horii Y, Matsui M. 2012. A comprehensive analysis of interaction and localization of Arabidopsis SKP1-like (ASK) and F-box (FBX) proteins. PLoS One 7:e50009.

Li Y, Zhang L, Li D, Liu Z, Wang J, Li X, Yang Y. 2016. The Arabidopsis F-box E3 ligase RIFP1 plays a negative role in abscisic acid signalling by facilitating ABA receptor RCAR3 degradation. Plant Cell Environ 39:571-582.
Lohmann D, Stacey N, Breuninger H, Jikumaru Y, Muller D, Sicard A, Leyser O, Yamaguchi S, Lenhard M. 2010. SLOW MOTION is required for within-plant auxin homeostasis and normal timing of lateral organ initiation at the shoot meristem in Arabidopsis. Plant Cell 22:335-348.
Nibau C, Gibbs DJ, Bunting KA, Moody LA, Smiles EJ, Tubby JA, Bradshaw SJ, Coates JC. 2011. ARABIDILLO proteins have a novel and conserved domain structure important for the regulation of their stability. Plant Mol Biol 75:77-92.
Potuschak T, Lechner E, Parmentier Y, Yanagisawa S, Grava S, Koncz C, Genschik P. 2003. EIN3-dependent regulation of plant ethylene hormone signaling by two arabidopsis F box proteins: EBF1 and EBF2. Cell 115:679-689.
Prigge MJ, Greenham K, Zhang Y, Santner A, Castillejo C, Mutka AM, O'Malley RC, Ecker JR, Kunkel BN, Estelle M. 2016. The Arabidopsis auxin receptor F-box proteins AFB4 and AFB5 are required for response to the synthetic auxin picloram. G3 (Bethesda) 6:1383-1390.
Risseeuw EP, Daskalchuk TE, Banks TW, Liu E, Cotelesage J, Hellmann H, Estelle M, Somers DE, Crosby WL. 2003. Protein interaction analysis of SCF ubiquitin E3 ligase subunits from Arabidopsis. Plant J 34:753-767.
Sepulveda-Garcia E, Rocha-Sosa M. 2012. The Arabidopsis F-box protein AtFBS1 interacts with 14-3-3 proteins. Plant Sci 195:36-47.
Stirnberg P, Furner IJ, Ottoline Leyser HM. 2007. MAX2 participates in an SCF complex which acts locally at the node to suppress shoot branching. Plant J 50:80-94.
Takahashi N, Kuroda H, Kuromori T, Hirayama T, Seki M, Shinozaki K, Shimada H, Matsui M. 2004. Expression and interaction analysis of Arabidopsis Skp1-related genes. Plant Cell Physiol 45:83-91.
van den Burg HA, Tsitsigiannis DI, Rowland O, Lo J, Rallapalli G, Maclean D, Takken FL, Jones JD. 2008. The F-box protein ACRE189/ACIF1 regulates cell death and defense responses activated during pathogen recognition in tobacco and tomato. Plant Cell 20:697-719.
Woo HR, Chung KM, Park JH, Oh SA, Ahn T, Hong SH, Jang SK, Nam HG. 2001. ORE9, an F-box protein that regulates leaf senescence in Arabidopsis. Plant Cell 13:1779-1790.
Xu L, Liu F, Lechner E, Genschik P, Crosby WL, Ma H, Peng W, Huang D, Xie D. 2002. The SCF(COI1) ubiquitin-ligase complexes are required for jasmonate response in Arabidopsis. Plant Cell 14:1919-1935.
Yasuhara M, Mitsui S, Hirano H, Takanabe R, Tokioka Y, Ihara N, Komatsu A, Seki M, Shinozaki K, Kiyosue T. 2004. Identification of ASK and clock-associated proteins as molecular partners of LKP2 (LOV kelch protein 2) in Arabidopsis. J Exp Bot 55:2015-2027.
Zhang X, Gou M, Liu CJ. 2013. Arabidopsis Kelch repeat F-box proteins regulate phenylpropanoid biosynthesis via controlling the turnover of phenylalanine ammonia-lyase. Plant Cell 25:4994-5010.
